# Schwannoma in the Upper Limbs

**DOI:** 10.1155/2013/167196

**Published:** 2013-09-04

**Authors:** Chris Yuk Kwan Tang, Boris Fung, Margaret Fok, Janet Zhu

**Affiliations:** Department of Orthopaedics & Traumatology, University of Hong Kong, 5/F, Professorial Block, Queen Mary Hospital, 102 Pokfulam Road, Hong Kong

## Abstract

Schwannomas are the commonest tumours of peripheral nerves. Despite the classical description that schwannomas are well encapsulated and can be completely enucleated during excision, a portion of them have fascicular involvement and could not be completely shelled out. A retrospective review for 8 patients was carried out over 10 years. 75% of schwannoma occurred over the distal region of upper limb (at elbow or distal to it). It occurs more in the mixed nerve instead of pure sensory or motor nerve. 50% of patients had mixed nerve involvement. Fascicular involvement was very common in schwannoma (75% of patients). Removal of the tumour with fascicles can cause functional deficit. At present, there is no method (including preoperative MRI) which can predict the occurrence of fascicular involvement; the authors therefore proposed a new system to stratify patients who may benefit from interfascicular nerve grafts. In this group of patients, the authors strongly recommend that the possibility and option of nerve graft should be discussed with patients prior to schwannoma excision, so that nerve grafting could be directly proceeded with patient consent in case there is fascicular involvement of tumour found intraoperatively.

## 1. Introduction

Schwannomas, also known as neurilemmoma, are benign tumours originating from Schwann cells along the course of a nerve. They are the commonest tumours of peripheral nerves, although the incidence in adults is only 5% [1] and upper limb schwannomas contribute 19% of them [2]. The growth of these tumours is slow, so they can remain as painless swellings for a few years before other symptoms appear [3]. There is a higher incidence in the flexor surface of the upper limb, since the concentration of nerve fibres is higher over that region [4].

Most of the schwannomas could be diagnosed clinically. Schwannomas are mobile in the longitudinal plane along the course of the involved nerve but not the transverse plane [5]. There is Tinel's sign (shooting paresthesia in the distribution of the involved nerve) upon percussion of the tumour [6] if the affected nerve is a sensory nerve or a mixed nerve.

## 2. Patients, Methods, and Results

We conducted a retrospective review for 8 adult patients with schwannoma, from June 2002 to November 2012. There are 2 men and 6 women, ranging from 20 to 88 years of age, with a mean age of 56 years old. All patients had excision done for the tumour and histopathological examination confirmed schwannoma.

12.5% (1 patient) had involvement of motor nerve, 37.5% (3 patients) had involvement of sensory nerve, and 50% (4 patients) had involvement of mixed nerve. The details of the patients are summarized in Table 1.

## 3. Discussion

In our case series, all patients with schwannoma involving sensory nerve or mixed nerve had positive Tinel's sign. This showed that a positive Tinel's sign carries a high predictive value (87.5% sensitivity) for schwannoma. The sites of schwannoma ranged from brachial plexus to dorsal branch of common digital nerve. In our case series, 75% (6 patients) of the tumour occurred at the level of elbow or distal to it, while 25% (2 patients) arose proximal to elbow. This may be because masses at distal upper limbs are easier to be noticed than the proximal ones. 50% (4 patients) had schwannoma affecting mixed nerves, 37.5% (3 patients) had pure sensory nerve involvement, and 12.5% (1 patient) had pure motor nerve involvement.

**Figure 1:**
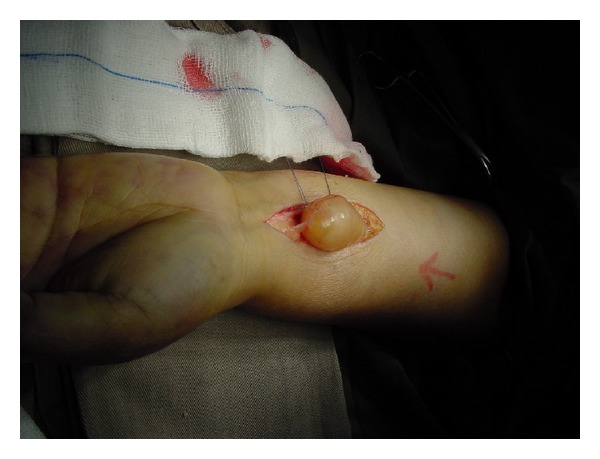
Schwannoma involving median nerve that can be completely enucleated.

Despite the conventional description [7] that most schwannoma can be completely enucleated without damaging the nerve because nerve fibres are displaced and do not penetrate the tumour (Figure 1), a large portion of them in our series had fascicular involvement. 75% (6 patients) involved nerve fascicles while 25% (2 patients) schwannoma could be completely enucleated without nerve fascicles involvement. The literature showed conflicting results regarding the function of nerve fascicles running through the tumour. Donner et al. [6] showed that there was no nerve action potential transmission through the tumour via these fascicles, suggesting that division of the affected fascicles will not induce neurological deficit. Yet a recent study [8] showed that 75% of patients had immediate neurological deficit after excision. This was believed to be due to the transection of fascicles that ran through the tumour. In our centre, electrical stimulations of the involved fascicles were used intraoperatively to assess the involvement.

Only one patient with nerve fascicles involvement underwent operation with left sural nerve graft. For patient number 3, although there was mixed nerve involvement, nerve graft was not done because of the patient's refusal. Her functional demand was not high due to her advanced age. For patient number 7, the main trunk of recurrent motor nerve was intact intraoperatively, and therefore nerve graft was not done. Nerve grafts had not been done for patients with pure sensory nerve involvement in our series. The majority of the pure sensory nerves are in the distal part of upper limb. As a result, only a small portion of area is affected which is usually clinically less significant because of the overlapping of sensory nerves. On the other hand, functional deficits are more disabling in the damage of motor nerve and mixed nerve (due to the presence of motor component), so the option of nerve graft would be discussed preoperatively with the patient. The authors believe that there will be distal neurological deficit caused by the excision of schwannoma with fascicular involvement (75% of patients (6 out of 8)). However, the small number of nerve grafts performed (1 out of 8 patients) in this study was accountable by multiple factors including patient preference and age of patient; the affected nerve was a distal sensory nerve. If the patient could not accept the risk of neurological deficit preoperatively, nerve grafts would be performed for all of the 6 patients with fascicular involvement. The lengths of stay in hospital for all eight patients were 3 days. The outcome of operation was dependent on the type of schwannoma. In patients who had complete enucleation of tumour, there were no complications. In patients with pure sensory nerve fascicular involvement, there was some numbness since nerve graft was not done. However, these were not clinically significant as all of them were distal sensory nerves. In patients with mixed nerve fascicular involvement, patient number 1 had partial sensory return to have protective sensation, but motor power had not recovered completely. Patient number 3 had mild sensory and motor deficit along the median nerve, but there did not have an impact on patients activities of living due to her age and patient preferred not for nerve graft preoperatively. In the only patient with pure motor fascicular involvement, there was no deficit as the main trunk of recurrent motor nerve was intact.

**Figure 2:**
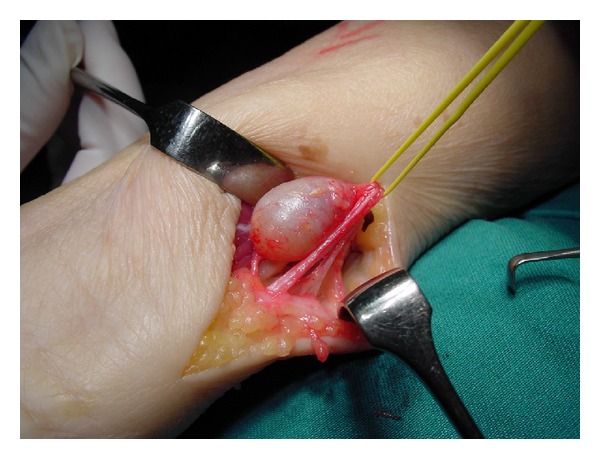
Schwannoma involving dorsal cutaneous branch of ulnar nerve which has fascicular involvement.

**Figure 3:**
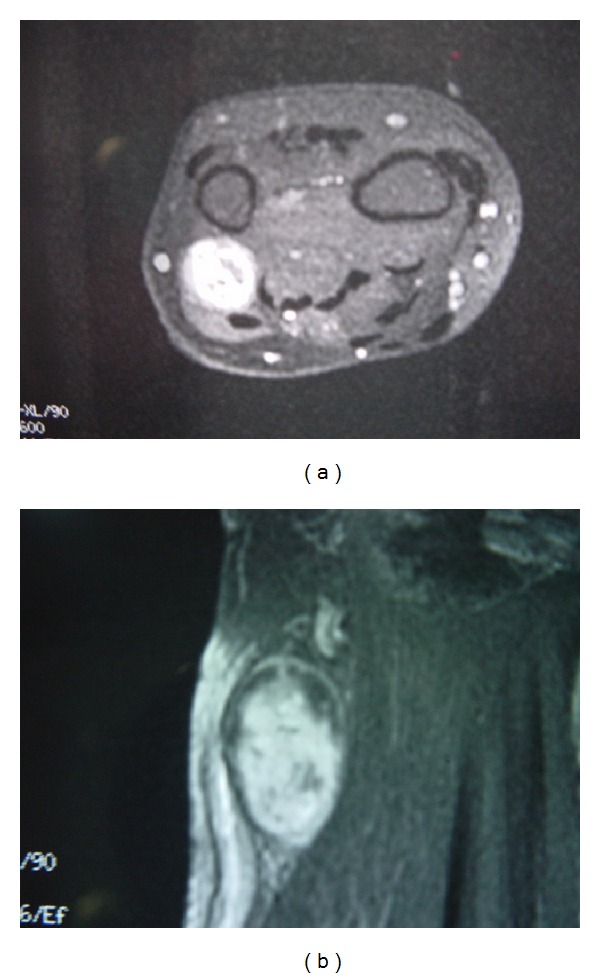
MRI showing schwannoma involving dorsal cutaneous branch of ulnar nerve, while the presence of fascicular involvement is not predictable in preoperative MRI.

In MRI, schwannomas are isointense to muscles in T1-weighted images and hyperintense in T2-weighted ones [9]. Features suggesting schwannoma include location in the region of a major nerve, depiction of nerve entering or exiting the mass, target sign (low signal intensity centrally (central fibrous components) and high signal intensity peripherally (peripheral myxomatous elements)), and fascicular sign (appearance of fascicular bundles) [10]. A portion of schwannoma has fascicular involvement (Figure 2). This group of tumours often cannot be completely dissected out from the involved fascicles. As a result, resection of fascicles becomes unavoidable during excision of the mass. This can lead to residual neurological deficit. Even though there are some radiological features that point to the diagnosis of schwannoma, MRI cannot predict whether the mass can be completely enucleated during the excision (Figure 3). Further studies should be carried out to look for specific features or imaging that can identify fascicular involvement preoperatively.

**Figure 4:**
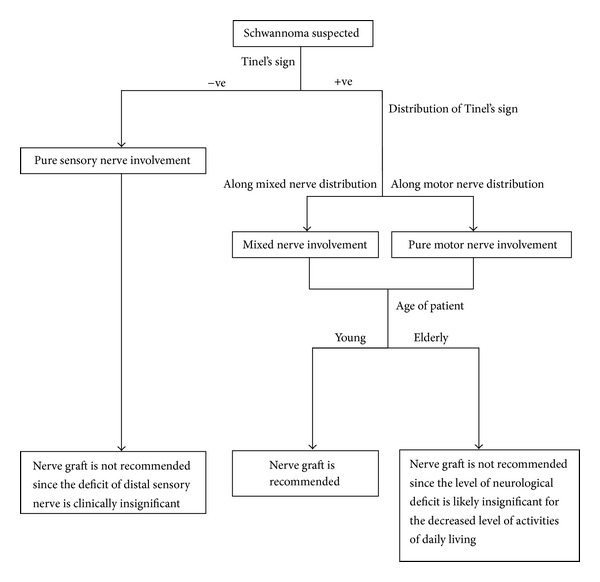
Management algorithm for schwannoma nerve graft.

Based on our case series, the authors proposed the following management algorithm for the decision of nerve graft in schwannoma with fascicular involvement (Figure 4). As mentioned earlier, fascicular involvement in schwannoma could not be identified preoperatively, but a consent is needed from the patient for nerve graft in order to spare the patient from a second operation. Therefore we recommend to use this algorithm preoperatively to stratify the patients who may need nerve graft in the operation. Surgeons should always discuss with the patient and prepare for nerve graft preoperatively for excision of mixed nerve schwannoma in young patients. Certainly, patient preference is also another decision factor.

In conclusion, schwannoma was an uncommon tumour, with only 8 cases for the past 10 years in our centre. It had a predilection over the distal region of upper limb (at elbow or distal to it), which might be explained by the fact that tumours at these sites are easier to be noticed by the patient. It occurs more in the mixed nerve instead of pure sensory or motor nerve. All these findings are consistent with the current literature. Two categories of schwannoma are found in our series. The first category involves well-encapsulated tumours in which they can be completely shelled out during the excision. As a result, fascicles are not involved. The second category consists of tumours with fascicular involvement. The tumour and the fascicles cannot be divided with naked eyes or microscopy during the excision, so a portion of the fascicles have to be excised with the tumour. Depending on the type of nerve involvement, functional deficit can result. At present, there has not yet been a reliable method to predict the presence of fascicular involvement by the tumour including preoperative MRI; the authors therefore proposed a new algorithm to preoperatively stratify patients who may benefit from interfascicular graft. For the group of patients who may benefit from interfascicular graft, the possibility for nerve graft should be discussed with the patient before the excision of schwannoma, and a consent should be obtained, in order to spare from a second operation for nerve graft if there is fascicular involvement.

## Figures and Tables

**Table 1:** 

No.	Age	Sex	Site	Nerve involved	Type	Nerve graft
1	25	F	Left axilla	Ulnar nerve	Fascicular involvement	Interfascicular nerve graft by left sural nerve
2	57	F	Left forearm	Median nerve	Complete enucleation	Nil
3	88	F	Left wrist	Median nerve	Fascicular involvement	Nil
4	48	M	Right wrist	Superficial branch of radial nerve (sensory)	Fascicular involvement	Nil
5	78	F	Right wrist	Dorsal cutaneous branch of ulnar nerve (sensory)	Fascicular involvement	Nil
6	49	F	Left infraclavicular fossa	Brachial plexus (posterior cord)	Complete enucleation	Nil
7	50	F	Right thenar eminence	Branch of right recurrent motor nerve	Fascicular involvement	Nil
8	53	M	Left middle finger	Left M/F ulnar digital nerve dorsal branch	Fascicular involvement	Nil
